# Cell–cell junction gene signatures as subtype-specific prognostic biomarkers in breast cancer

**DOI:** 10.1371/journal.pone.0352254

**Published:** 2026-06-29

**Authors:** Hayato Ishii, Kyoka Nishiyama, Ayaka Nakahara, Shoma Tamori, Shigeo Ohno, Kazunori Sasaki, Kazunori Akimoto

**Affiliations:** 1 Department of Medicinal and Life Sciences, Faculty of Pharmaceutical Sciences, Tokyo University of Science, Tokyo, Japan; 2 Research Division of Medical Data Science, Research Institute for Science and Technology, Tokyo University of Science, Chiba, Japan; 3 Laboratory of Cancer Biology, Institute for Diseases of Old Age, Juntendo University School of Medicine, Tokyo, Japan; The University of Queensland Faculty of Medicine, AUSTRALIA

## Abstract

Cell–cell junctions (CCJs) are essential for maintaining epithelial integrity, and adhesion-related molecules have long been implicated in breast cancer progression. However, the subtype-specific prognostic significance of CCJ-related gene expression patterns within individual intrinsic breast cancer subtypes has not been systematically characterized. We analyzed 179 genes annotated to the Gene Ontology term “cell–cell junction organization” (GO:0045216) across intrinsic breast cancer subtypes using the METABRIC and The Cancer Genome Atlas (TCGA) datasets. Subtype-specific prognostic CCJ genes were identified using multivariate Cox proportional hazards models for disease-specific survival and integrated into CCJ gene expression signatures. The prognostic performance was validated in an independent cohort (SCAN-B). Elevated CCJ signature scores were associated with poorer survival across subtypes, with particularly strong effects in Luminal B (LumB) and Basal-like (Basal) tumors. Person-year analyses indicated that high CCJ scores predicted an increased incidence of early recurrence (0–5 years) in these aggressive subtypes. Pathway enrichment analyses revealed that high-score tumors exhibited upregulation of extracellular matrix organization and matrisome-related pathways. Single-cell RNA sequencing further demonstrated that LumB CCJ genes (e.g., *PARD6B*, *CDH3*) were predominantly expressed in tumor epithelial cells, whereas the Basal CCJ signature reflected contributions from epithelial (e.g., *MARVELD2*) and endothelial (e.g., *RAMP2*) cells. Collectively, CCJ signatures stratify prognosis and capture subtype-specific cellular and microenvironmental features in breast cancer.

## Introduction

Breast cancer is the most prevalent malignancy among women worldwide, and its incidence and mortality continue to pose clinical challenges [1]. Breast cancer is now widely recognized not as a single disease but as a biologically heterogeneous group of malignancies comprising multiple molecular subtypes defined by receptor status and gene expression profiles [2]. In particular, the clinically implemented PAM50 intrinsic subtyping system, including the Claudin-low category, classifies breast cancer into six major subtypes: Luminal A (LumA), Luminal B (LumB), Her2-enriched (Her2), Normal-like (Normal), Claudin-low, and Basal-like (Basal) [2–6]. These subtypes are closely associated with clinicopathological features such as hormone receptor status, Her2 expression, and proliferative activity. Because each subtype exhibits distinct biological behavior, therapeutic sensitivity, and outcome, intrinsic subtyping has become the basis of precision medicine [3,4,7–12]. Despite these advances, substantial heterogeneity in clinical outcomes persists among patients of the same intrinsic subtype [13,14]. Furthermore, clinically implemented multigene assays such as Oncotype DX, MammaPrint, and EndoPredict have improved risk stratification and assisted treatment decision-making in hormone receptor–positive breast cancer [15,16]. However, these assays rely on predefined gene panels and are mainly designed for patients with early-stage luminal disease receiving endocrine therapy (e.g., tamoxifen). Consequently, their ability to fully capture biological heterogeneity within subtypes or treatment-specific vulnerabilities remains limited, underscoring the need for biomarkers that complement the current signatures. Therefore, identifying novel biomarkers that complement existing classifications and enable more precise prognostic stratification remains an urgent priority.

Disruption and aberrant reconstruction of cell–cell adhesion, which normally maintains epithelial homeostasis, represent fundamental processes in cancer initiation and progression [17]. Cell–cell adhesion is mediated by the coordinated activity of multiple junctional complexes—including adherens junctions, tight junctions, gap junctions, and desmosomes [18–21]—whose dysfunction contributes directly to uncontrolled proliferation, invasion, and metastatic dissemination of tumor cells [17,22,23]. Previously, research in this area has focused on individual adhesion molecules. For instance, although loss of E-cadherin (CDH1) expression was traditionally considered a key trigger for invasion, it has been shown to play a critical role in tumor cell survival during metastasis [24]. Similarly, aberrant expression of claudins has been linked to Claudin-low subtype of breast cancer [5]. Gene family–level studies, such as comprehensive analyses of the cadherin family, have further demonstrated that the simultaneous dysregulation of multiple cadherins is associated with poor prognosis [25]. More recently, broader approaches have been proposed; for instance, Lv et al. developed a prognostic model based on a wide range of “cell adhesion-related genes” (CARGs), which also included genes involved in cell-extracellular matrix adhesion [26].

Nevertheless, several important challenges remain. First, although the study by Lv et al. provided a broad overview of adhesion-related processes, it did not specifically focus on cell–cell adhesion, a distinct biological mechanism that represents an early and critical step in the loss of intercellular cohesion during tumor invasion and metastasis. Second, most previous large-scale analyses have treated breast cancer as a single entity, thereby overlooking clinically established subtype-specific differences in prognosis and therapeutic response. Consequently, the precise role of the biological process “cell–cell junction organization” within individual breast cancer subtypes—and its relationship to patient outcome—remains insufficiently understood. Moreover, the prognostic implications of cell–cell adhesion molecules are often paradoxical. In some cancer types, high expression of adhesion molecules such as claudins and cadherins correlates with aggressive behavior and poor prognosis [24,27,28], whereas in other contexts, the same molecules are associated with favorable outcomes [28,29]. These contradictory findings have hindered a unified understanding of the biological and clinical significance of cell–cell junction–associated genes in cancer progression. To address this complexity, we aimed to develop an integrative framework that captures the collective behavior of genes involved in cell–cell junction (CCJ) organization, rather than focusing on individual molecules. By constructing and validating CCJ gene expression signatures across intrinsic breast cancer subtypes, we sought to clarify how coordinated expression of adhesion-related genes reflects subtype-specific tumor biology and prognosis. Specifically, we performed a comprehensive analysis of genes annotated to the Gene Ontology term “cell–cell junction organization” (GO:0045216), identified subtype-specific prognostic genes using large-scale transcriptomic datasets from the METABRIC and The Cancer Genome Atlas (TCGA) cohorts, and constructed CCJ signatures for each subtype. These signatures were subsequently validated in the Sweden Canceromics Analysis Network-Breast (SCAN-B) cohort to assess their robustness and reproducibility. Furthermore, we examined whether their prognostic associations were influenced by histological subtype or canonical EMT-related genes, including *CDH1*, and assessed the temporal dynamics of recurrence risk associated with these signatures. Finally, by integrating differential gene expression and single-cell RNA-seq data, we investigated the molecular characteristics and cell-type–specific expression patterns underlying each subtype-specific CCJ signature. Through this approach, we present a systems-level framework that unifies the complex and sometimes contradictory roles of cell–cell junction–related genes in breast cancer progression and prognosis.

## Materials and methods

### Ethics statement

The study involved secondary analyses of publicly available, de-identified datasets, including METABRIC, TCGA, SCAN-B, and publicly accessible single-cell RNA-seq data. Because all data were fully anonymized prior to access and no direct interaction with human participants occurred, ethical approval from an institutional review board or ethics committee was not required. The requirement for informed consent was waived as all data were fully anonymized prior to acquisition.

### METABRIC dataset

The METABRIC dataset (n = 2,509) [30,31] was downloaded from cBioPortal (https://www.cbioportal.org/) [32,33] on September 2, 2024. Samples with available data for DSS, mRNA expression, and PAM50 subtype information (n = 1,479) were included in prognostic analyses. Based on PAM50 classification, tumors were categorized as LumA (n = 475), LumB (n = 348), Her2 (n = 175), Normal (n = 119), Basal (n = 184), and Claudin-low (n = 178). Additionally, for the recurrence risk analyses, samples with available relapse-free survival (RFS) data (n = 1,825) were used, including LumA (n = 699), LumB (n = 475), Her2 (n = 224), Basal (n = 209), and Claudin-low (n = 218) subtypes.

### TCGA dataset

The TCGA Pan-Cancer Atlas breast cancer dataset (n = 1,084) [34] was downloaded from cBioPortal (https://www.cbioportal.org/) [32,33] on November 19, 2024. Samples with available DSS, mRNA expression, and PAM50 subtype information (n = 962) were included. Tumors were stratified based on PAM50 classification: LumA (n = 491), LumB (n = 192), Her2 (n = 77), Normal (n = 35), and Basal (n = 167). Additionally, for the recurrence risk analyses, samples with available DFS data (n = 823) were analyzed, including LumA (n = 432), LumB (n = 172), Her2 (n = 66), and Basal (n = 153) subtypes.

### The SCAN-B dataset

The SCAN-B cohort (n = 3,678) [35] was downloaded from the Gene Expression Omnibus (GEO) (https://www.ncbi.nlm.nih.gov/geo/) under accession number GSE96058. Samples with available overall survival (OS), mRNA expression, and PAM50 classification data (n = 3,052) were included. Tumors were stratified based on PAM50 classification: LumA (n = 1,657), LumB (n = 729), Her2-enriched (n = 327), and Basal (n = 339). For each gene, mRNA expression values were z-score normalized by subtracting the mean and dividing by the standard deviation across all samples for subsequent analyses.

### Prognostic analysis

DSS was analyzed using Kaplan-Meier survival curves, log-rank (Cochran-Mantel-Haenszel) tests, and multivariate Cox proportional hazards regression analyses, as previously described [36,37]. Patients were stratified into high- and low-expression groups based on z-score-normalized expression of individual genes or CCJ signature scores. The optimal cutoff values were determined using receiver operating characteristic (ROC) curve analysis for DSS, maximizing the Youden index. Age at diagnosis was included as a confounding factor in all multivariate Cox models.

### Identification of prognostic cell–cell adhesion genes

To identify cell–cell adhesion molecules with prognostic relevance in each subtype, a gene list was obtained from the Gene Ontology term “cell–cell junction organization” (GO:0045216) (S1 Table). In each intrinsic subtype within the discovery datasets (METABRIC and TCGA), patients were stratified into high- and low-expression groups for each gene using the same z-score-based approach described above. Because the TCGA dataset does not include a distinct Claudin-low subtype, genes identified as prognostic in either the Basal or Claudin-low subtypes of the METABRIC cohort were intersected with genes identified in the Basal subtype of the TCGA dataset. Genes showing a statistically significant association with DSS in both datasets (*p* < 0.05, with 95% CI for HRs not crossing 1) were defined as robust subtype-specific prognostic genes. These 179 genes were used only as an initial candidate pool for subtype-specific prognostic screening in the discovery cohorts and were not used directly as the input for the downstream GO enrichment analysis.

### Calculation of the CCJ signature score

Subtype-specific CCJ signatures were constructed based on the selected prognostic genes, as previously described [38]. For each patient, the CCJ signature score was calculated using the following formula:


$$\begin{array}{c} CCJSignatureScore=\frac{\sum_{i=\text{1}}^{n}Z_{poor,i}-\sum_{j=\text{1}}^{m}Z_{favorable,j}}{n+m} \end{array}$$

where $\begin{array}{c} Z_{poor,i} \end{array}$ represents the z-score-normalized expression value of the i-th gene associated with poor prognosis, $Z_{favorable,j}$ represents the z-score-normalized expression value of the j-th gene associated with favorable prognosis, and *n* and *m* denote the total numbers of poor- and favorable-prognosis genes, respectively. Using these CCJ signature scores, patients in the discovery cohorts (METABRIC, TCGA) and the validation cohort (SCAN-B) were stratified into high- and low-score groups. Survival analyses, including Kaplan-Meier estimation and multivariate Cox proportional hazards modeling, were then performed. In the SCAN-B dataset analysis, the Her2 signature gene *AJM1* and the LumA signature genes *AFDN* and *INAVA* were not available (NA) and were therefore excluded from the corresponding CCJ signature score calculations.

### Histological subtype analysis

To examine whether the prognostic associations of the CCJ signatures were influenced by histological subtype, we performed a histology-focused analysis in the METABRIC and TCGA cohorts. Histological annotation was obtained from the dataset-specific pathology fields: the *Tumor.Other.Histologic.Subtype* column in METABRIC and the *Tumor.Type* column in TCGA. Because annotation schemes differed between datasets, histological categories were harmonized into simplified groups prior to analysis.

In METABRIC, cases annotated as “Lobular” were classified as invasive lobular carcinoma (ILC), and cases annotated as “Ductal/NST” were classified as non-ILC in the main comparative analyses. For descriptive analyses, “Mixed” was retained as a separate category, whereas all remaining annotations (including “Medullary,” “Metaplastic,” “Mucinous,” “Tubular/ cribriform,” “Other,” and “NA”) were grouped as “Other”. In TCGA, cases annotated as “Infiltrating Lobular Carcinoma” were classified as ILC, cases annotated as “Infiltrating Ductal Carcinoma” were classified as non-ILC, and “Mixed Histology (NOS)” was treated as “Mixed,” and all other categories (including “Breast Invasive Carcinoma,” “Infiltrating Carcinoma [NOS],” “Medullary Carcinoma,” “Metaplastic Carcinoma,” and “Mucinous Carcinoma,”) were grouped as “Other.”

Histological composition within each intrinsic subtype was summarized using 100% stacked bar plots. For the primary comparative analyses, only ILC and non-ILC cases were included, excluding “Mixed” and “Other” categories. CCJ signature scores were compared between ILC and non-ILC groups within each subtype using subtype-matched CCJ signature. Differences in CCJ signature scores were assessed using the Wilcoxon rank-sum test within each subtype, followed by Benjamini–Hochberg correction for multiple testing. To determine whether the association between CCJ signature classification and survival was independent of histological subtype, multivariable Cox proportional hazards models were fitted including CCJ signature class (high vs low), age at diagnosis, and simplified histology (ILC vs non-ILC) as covariates. Analyses were restricted to cases with complete data for survival, age, histology, and CCJ signature classification. Hazard ratios (HRs) with 95% confidence intervals (CIs) were estimated, and results were summarized using forest plots for each cohort.

### Prognostic evaluation of EMT-related CCJ genes and *CDH1*

To further examine the relationship between the CCJ signatures and canonical epithelial–mesenchymal transition (EMT)-related genes, we assessed the overlap between the Gene Ontology (GO) term GO:0045216 (“cell–cell junction organization”) gene set and the HALLMARK_EPITHELIAL_MESENCHYMAL_TRANSITION gene set obtained from the Molecular Signatures Database (MSigDB) [39,40]. Because *CDH1*, a key epithelial marker with a well-established role in EMT, is not included in the HALLMARK EMT gene set, it was additionally incorporated into this analysis. Multivariable Cox proportional hazards analyses were then performed for the overlapping genes and *CDH1* in the METABRIC and TCGA cohorts using the same subtype-specific framework described above. Hazard ratios (HRs) with 95% confidence intervals (CIs) were estimated, and statistical significance was defined as p < 0.05 with CIs not crossing 1.

### Leave-one-gene-out sensitivity analysis of the CCJ signature

To assess the robustness of the CCJ signatures, we performed a leave-one-gene-out sensitivity analysis. For each subtype-specific signature, the original CCJ signature score was calculated as described above and then recalculated iteratively by omitting one gene at a time from the signature. For each recalculated signature, patients were reclassified into high- and low-score groups using receiver operating characteristic (ROC) analysis with the Youden index. Age-adjusted multivariable Cox proportional hazards models were then fitted using the same cohort-specific procedures as in the primary analyses. Hazard ratios (HRs) with 95% confidence intervals (CIs) derived from the original and leave-one-gene-out models were compared to evaluate the stability of the prognostic associations. To facilitate interpretation of potential redundancy among equally weighted genes, a subtype-specific integrated rank table was additionally generated from the discovery cohorts (S4 Table). This table was not used to modify the leave-one-gene-out models but was used descriptively to summarize the consistency of gene-level prognostic contributions across cohorts.

### Gene-number downsampling analysis of the CCJ signature

To evaluate how many genes were required for each CCJ signature to retain prognostic information, we performed a gene-number downsampling analysis. To generate reduced signatures, genes were prioritized using a subtype-specific integrated rank table derived from the discovery cohorts (S4 Table). Gene-level prognostic ranks were defined based on the strength and consistency of associations with survival outcomes in each cohort. Briefly, within each subtype, gene-level prognostic ranks were calculated separately in the METABRIC and TCGA datasets and then averaged to obtain a mean rank. For the Basal context, METABRIC Basal and Claudin-low ranks were considered separately, and the better METABRIC rank was selected before averaging with the TCGA Basal rank. Reduced signatures were constructed by selecting top-ranked genes within each subtype. For each reduced gene set, CCJ signature scores were calculated using the formula described above, and patients were reclassified into high- and low-score groups using receiver operating characteristic (ROC) analysis with the Youden index. Age-adjusted multivariable Cox proportional hazards models were then fitted using the same cohort-specific procedures as in the primary analyses. In the SCAN-B dataset, only genes with available expression values were retained, and gene sets with fewer than two matched genes were excluded from analysis. Hazard ratios (HRs) with 95% confidence intervals (CIs) derived from the original and downsampled signatures were compared to evaluate the stability of prognostic associations as gene number decreased.

### Recurrence risk analysis

Recurrence risk was evaluated using person-years approach, as previously described [36]. Patients were stratified into high- and low-score groups based on their CCJ signature scores, using optimal cutoff values determined by ROC analysis. Follow-up time was divided into predefined intervals to capture time-dependent recurrence patterns. For the METABRIC dataset, using RFS as the endpoint, intervals were defined as 0–5, 5–10, 10–15, and >15 years. For the TCGA dataset, using DFS as the endpoint, intervals were defined as 0–5, 5–10, and >10 years, reflecting the available follow-up distribution. Recurrence incidence rates were calculated as the number of events per person-year within each interval using the pyears function from the R survival package. To compare the risk between high- and low-score groups, the incidence rate ratios (IRRs) with corresponding 95% CIs were estimated. Statistical significance was assessed using the Poisson exact test. To account for multiple comparisons across time intervals, *P*-values were adjusted using the Holm method, and adjusted *p* < 0.05 was considered statistically significant.

### Differential gene expression and functional enrichment analysis

To characterize biological differences associated with CCJ signature scores, differential gene expression (DEG) analysis was performed between high- and low-score groups in the TCGA and SCAN-B datasets. DEG analysis was restricted to the TCGA and SCAN-B cohorts because both datasets were generated using RNA-seq-based transcriptomic profiling, thereby reducing platform-related heterogeneity in fold-change-based comparisons; the METABRIC dataset was not included in this step. Because the available expression matrices differed in format, DEG analysis was conducted using different statistical frameworks: DESeq2 was applied to the TCGA count-like expression matrix, whereas limma was applied to the transformed SCAN-B expression matrix. As an internal positive control, we additionally evaluated whether each subtype-specific CCJ signature gene (i) was present in the expression matrix, (ii) passed expression filtering, and (iii) was identified as a significant DEG under the predefined criteria. Genes that were significantly and consistently altered in both cohorts (fold change [FC] > 2 or [FC] < 0.5, *p* < 0.05) were extracted. For commonly upregulated genes identified in the LumB and Basal CCJ signatures, GO enrichment analysis was performed using Metascape (http://metascape.org) [41] to identify associated biological processes and molecular functions.

### Single-cell RNA-seq analysis of breast cancer samples

Publicly available single-cell RNA sequencing (scRNA-seq) data from a human breast cancer atlas [42] were obtained from the Gene Expression Omnibus (GEO; accession number GSE176078). Raw gene expression count matrices and associated metadata were used as input. All analyses were performed using the Seurat package (version 5.3.0). A Seurat object was constructed from the count matrices, followed by quality control (QC) filtering to remove low-quality cells. Cells with fewer than 200 detected genes (nFeature_RNA) or cells with mitochondrial transcript content exceeding 20% were excluded. To focus on untreated tumor microenvironments, cells from patients with a history of therapy (patient IDs: CID3963, CID4066, CID4398, CID4513, CID4523) were excluded from downstream analyses. The final cohort comprised 21 untreated patients, stratified by clinical subtype as follows: ER+ (n = 10), Her2+ (n = 3), Her2 + /ER+ (n = 1), and TNBC (n = 7).

### Data normalization, integration, and clustering

Following QC, gene expression data were log-normalized using the NormalizeData function. The 2,000 most highly variable genes were identified using the variance-stabilizing transformation (vst) method implemented in FindVariableFeatures. Data were then scaled using ScaleData, and principal component analysis (PCA) was performed for dimensionality reduction. To correct for batch effects attributed to inter-patient variation, Harmony integration was applied to the first 50 principal components, using patient identity as the batch covariates. Harmony-corrected embeddings were used for all downstream analyses. For visualization, Uniform Manifold Approximation and Projection (UMAP) was conducted using the first 30 Harmony dimensions. Cell clustering was performed on the same dimensions by constructing a shared nearest neighbor (SNN) graph with FindNeighbors, followed by Louvain community detection using FindClusters at a resolution of 0.8.

### Cell type identification and annotation

Cell types were identified using a combination of automated and manual annotation approaches based on established marker gene expression. For automated annotation, the SingleR R package was employed using the Human Primary Cell Atlas reference dataset, obtained from the celldex package. The Seurat object was first converted into a SingleCellExperiment object, after which the SingleR() function was applied to assign the most probable cell type label and an associated confidence score to each cell. To validate and supplement the automated annotations, the specific expression patterns of known marker genes were visualized using the DotPlot function. The marker genes used for cell type identification were as follows: epithelial cells (*EPCAM*, *KRT18)*, T/NK cells (*PTPRC*, *CD3D*, *NKG7, KLRD1*), B-lineage cells (*MS4A1*, *JCHAIN)*, myeloid cells (*CD68*, *CD14*, *ITGAX*), fibroblasts (*COL1A1*, *DCN*, *PDGFRB*), and endothelial cells (*PECAM1*, *VWF*). Final cell type annotations were determined by integrating both results. Specifically, the primary cell type assigned to each cluster by SingleR was first reviewed and then cross-referenced with the specific expression patterns of the marker genes, resulting in a final cell type label assigned to each cluster.

### Cell type- and subtype-specific expression analysis of the CCJ signature

Using the cell type-annotated Seurat object, expression patterns of prognostic CCJ signature genes were evaluated in a subtype-specific manner. Cells corresponding to TNBC and Her2 + /ER+ subtypes were extracted from the untreated patient cohort, and separate Seurat subset objects were generated for each subtype. Expression of individual genes within the Basal CCJ signature (analyzed in TNBC samples) and the LumB CCJ signature (analyzed in Her2 + /ER+) was visualized using dot plots and UMAP-based feature plots generated with the FeaturePlot function. These visualizations enabled assessment of the specific cell populations contributing to CCJ gene expression within the tumor microenvironment of each subtype. Genes with undetected expression were displayed in gray across all cells to facilitate clear distinction.

### Visualization of results

Overlap among prognostic genes across breast cancer subtypes was visualized using UpSet plots generated with the UpSetR package in R. Genes consistently associated with prognosis in both the METABRIC and TCGA datasets were additionally illustrated using Venn diagrams constructed with the VennDiagram package. Results from multivariate Cox proportional hazards analyses were visualized using forest plots generated with the forestplot package in R.

### Statistical evaluation

All statistical analyses were performed using R software version 4.5.1 (R Core Team, R Foundation for Statistical Computing, Vienna, Austria).

## Results

**Fig 1:**
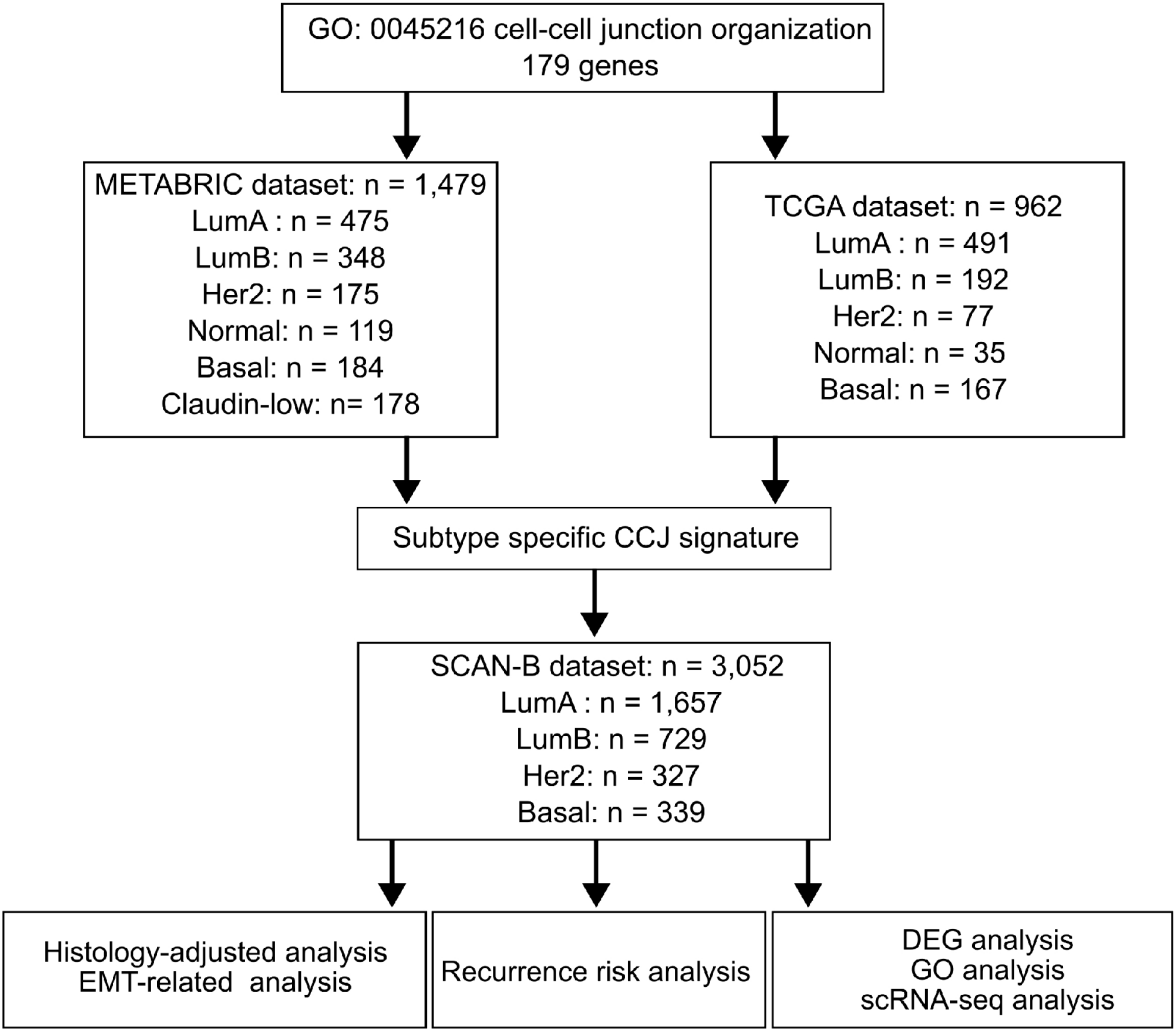
Overview of the study workflow. The flowchart of the study methodology.

To elucidate the prognostic relevance of genes involved in CCJ organization in breast cancer, we implemented a stepwise analytical framework integrating large-scale transcriptomic datasets and multi-level validation (Fig 1). First, disease-specific survival (DSS)–based multivariate Cox proportional hazards analyses were performed for all genes annotated under the Gene Ontology term “cell–cell junction organization” (GO:0045216) using two independent discovery cohorts, METABRIC and TCGA. Analyses were stratified by PAM50 intrinsic molecular subtypes, including the Claudin-low group. Subtype-specific prognostic genes were defined as those consistently identified across both datasets. Next, prognostic genes within each subtype were integrated to construct CCJ gene expression signatures, designed to capture the coordinated transcriptional behavior of multiple junctional components rather than isolated genes. The prognostic performance of each CCJ signature was evaluated using Kaplan-Meier survival analysis and multivariate Cox proportional hazards models in both the discovery and an independent validation cohort (SCAN-B). Furthermore, person-year analyses were performed to assess the recurrence risk.

To investigate the biological context underlying these signatures, we further compared differential gene expression profiles between high- and low-signature-score tumors in the TCGA and SCAN-B datasets. Finally, we examined cell-type–specific expression patterns of key genes in the LumB and Basal CCJ signatures using single-cell RNA-seq data from breast cancer patients (GSE176078). This multi-layered analytical approach enabled systematic characterization of the subtype-dependent prognostic impact and biological heterogeneity of CCJ-associated signaling in breast cancer.

### DSS-based multivariate Cox analyses of “cell–cell junction organization”–related genes across PAM50 subtypes

We first investigated the prognostic impact of genes involved in cell–cell junction organization across intrinsic breast cancer subtypes. DSS-based multivariate Cox proportional hazards analyses were performed for 179 genes annotated to GO:0045216 (S1 Table) in METABRIC and TCGA cohorts, adjusting for patient age as a covariate. Analyses were stratified by PAM50 subtypes, including the Claudin-low group, to account for subtype-specific biological differences.

**Fig 2:**
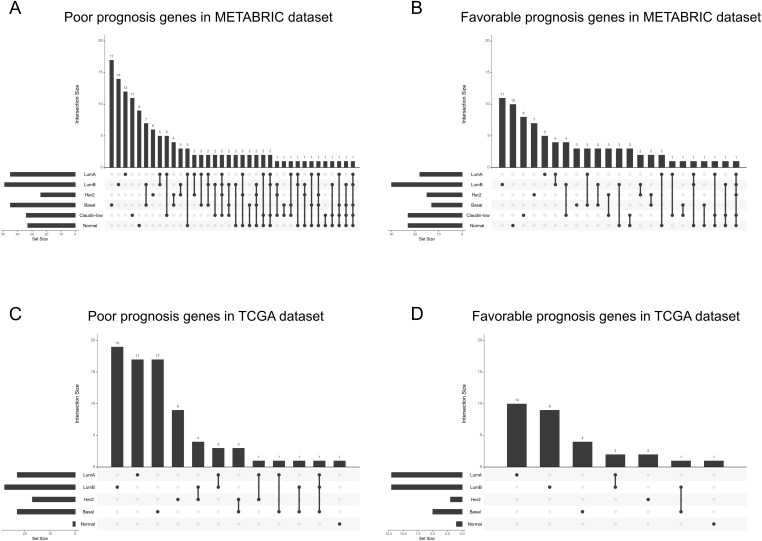
Prognostic landscape of cell–cell junction organization genes across breast cancer subtypes. Multivariate Cox proportional hazards analyses were performed for 179 genes annotated under the Gene Ontology term “cell–cell junction organization” (GO:0045216) to evaluate associations with DSS, adjusted for patient age. UpSet plots display the number of genes for which high expression was significantly associated with **(A)** poor prognosis and **(B)** favorable prognosis in the METABRIC dataset, and **(C)** poor prognosis and **(D)** favorable prognosis in the TCGA dataset. The vertical bars represent the number of significant genes shared among the indicated subtypes, highlighting the subtype-specific nature of prognostic associations.

**Fig 3:**
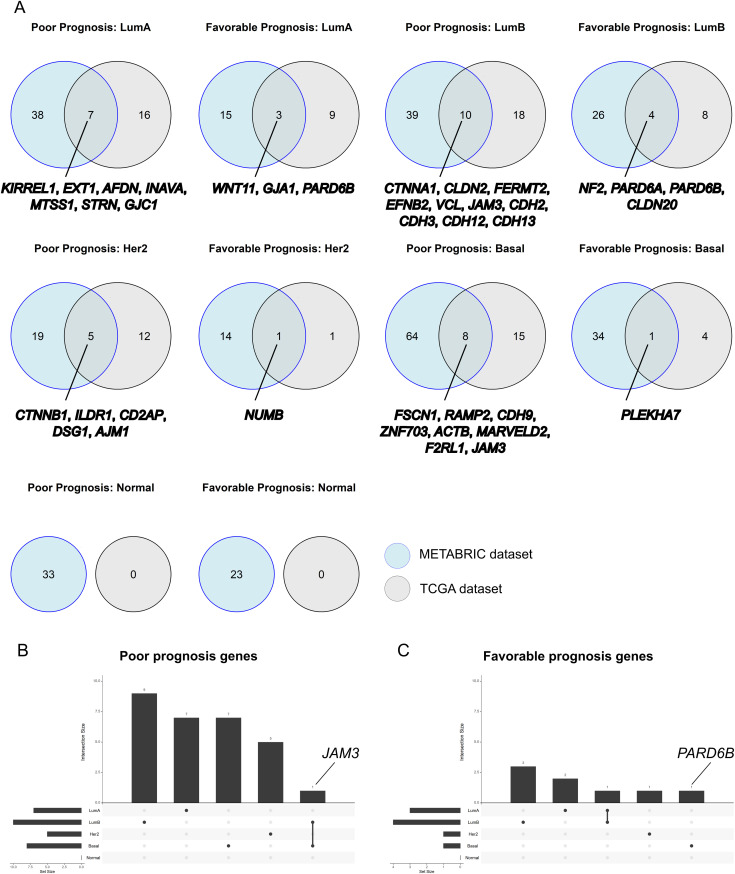
Identification of robust subtype-specific prognostic CCJ genes shared between METABRIC and TCGA datasets. **(A)** Venn diagrams illustrating the overlap of significant prognostic genes between the METABRIC (blue) and TCGA (grey) datasets for each molecular subtype. Intersections represent robust prognostic genes consistently identified in both independent cohorts. No common prognostic genes were identified for the Normal subtype. **(B)** UpSet plot displaying the intersection of robust poor-prognosis genes across different subtypes (e.g., overlap between LumB and Basal). **(C)** UpSet plot displaying the intersection of robust favorable-prognosis genes across different subtypes. These plots visualize the degree of subtype-specificity among the validated prognostic genes.

In both datasets, a substantial number of genes demonstrated significant associations with DSS, and these associations were largely subtype-specific, indicating that the prognostic relevance of CCJ-related genes differs markedly between molecular subtypes (Fig 2A–2D). Notably, no single gene was prognostic across all subtypes in either cohort, underscoring the heterogeneity of CCJ-related pathways in breast cancer progression. These findings provided the rationale for constructing subtype-specific CCJ gene expression signatures to capture coordinated transcriptional programs underlying subtype-dependent prognosis. To identify robust subtype-specific prognostic genes, we focused on genes consistently associated with DSS in both datasets. Genes associated with poor prognosis across both datasets included seven genes in LumA (*KIRREL1*, *EXT1*, *AFDN*, *INAVA*, *MTSS1*, *STRN*, *GJC1*), ten in LumB (*CTNNA1*, *CLDN2*, *FERMT2*, *EFNB2*, *VCL*, *JAM3*, *CDH2*, *CDH3*, *CDH12*, *CDH13*), five in Her2 (*CTNNB1*, *ILDR1*, *CD2AP*, *DSG1*, *AJM1*), and eight in Basal (*FSCN1*, *RAMP2*, *CDH9*, *ZNF703*, *ACTB*, *MARVELD2*, *F2RL1*, *JAM3*) (Table 1 and Fig 3A). Conversely, genes consistently associated with favorable prognosis included three genes in LumA (*WNT11*, *GJA1*, *PARD6B*), four in LumB (*NF2*, *PARD6A*, *PARD6B*, *CLDN20*), one in Her2 (*NUMB*), and one in Basal (*PLEKHA7*) (Table 1 and Fig 3A). No common prognostic genes were identified for the Normal subtype across both datasets. These findings suggest that CCJ-related genes may have a more limited or less consistent association with tumor progression and prognosis in the Normal subtype compared with other intrinsic subtypes. Although most prognostic genes were subtype-specific, *JAM3* represented a notable exception, being associated with poor prognosis in both LumB and Basal subtypes (Fig 3B). Similarly, *PARD6B* emerged as a favorable prognostic gene shared between LumA and LumB subtypes (Fig 3C). Among the representative subtype-specific genes selected for subsequent biological interpretation, *INAVA* was reproducibly associated with poor prognosis in LumA tumors, *CLDN2* and *FERMT2* were reproducibly associated with poor prognosis in LumB tumors, and *NUMB* was the sole gene reproducibly associated with favorable prognosis in Her2 tumors (Table 1 and Fig 3A). These genes were therefore considered representative subtype-linked components of the CCJ signatures and are discussed further below in the context of established breast cancer biology.

**Table 1:** Subtype-specific prognostic CCJ signature genes in breast cancer.

Subtype	Genes associated with poor prognosis	Genes associated with favorable prognosis
LumA	*KIRREL1*, *EXT1*, *AFDN*, *INAVA*, *MTSS1*, *STRN*, *GJC1*	*WNT11*, *GJA1*, *PARD6B*
LumB	*CTNNA1*, *CLDN2*, *FERMT2*, *EFNB2*, *VCL*, *JAM3*, *CDH2*, *CDH3*, *CDH12*, *CDH13*	*NF2*, *PARD6A*, *PARD6B*, *CLDN20*
Her2	*CTNNB1*, *ILDR1*, *CD2AP*, *DSG1*, *AJM1*	*NUMB*
Basal	*FSCN1*, *RAMP2*, *CDH9*, *ZNF703*, *ACTB*, *MARVELD2*, *F2RL1*, *JAM3*	*PLEKHA7*

List of genes significantly associated with poor or favorable prognosis in four clinical subtypes: LumA, LumB, Her2, and Basal. Prognostic associations were determined based on the consensus between METABRIC and TCGA datasets.

### Prognostic significance of CCJ signatures in the discovery cohorts

**Fig 4:**
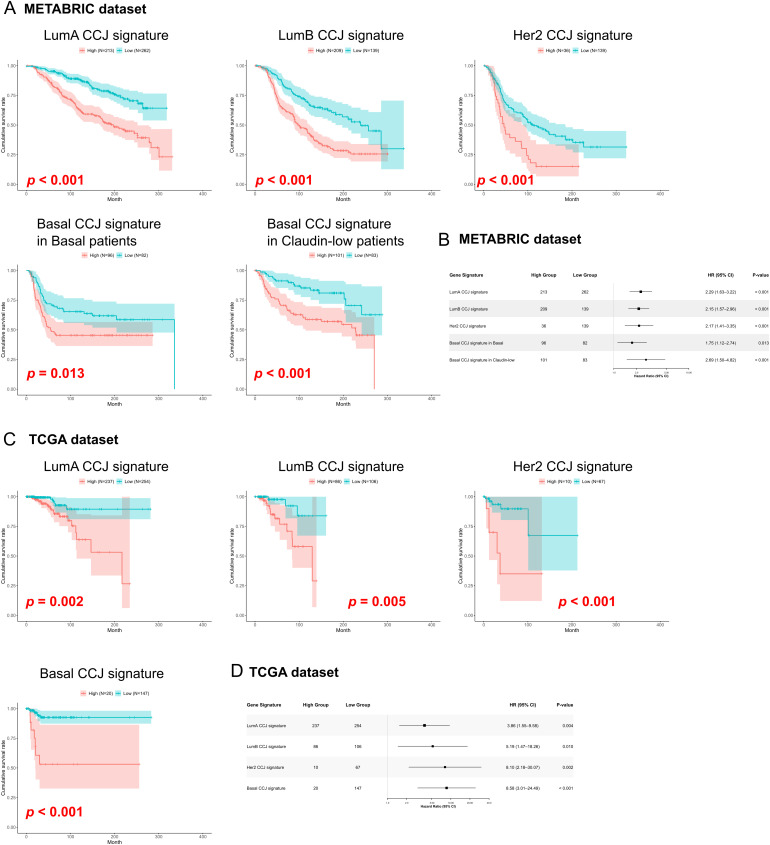
Prognostic significance of subtype-specific CCJ signatures in METABRIC and TCGA datasets. Kaplan-Meier survival analysis and multivariate Cox proportional hazards regression were performed to evaluate associations between CCJ signature scores and DSS. **(A)** Kaplan-Meier curves for the METABRIC dataset. Patients were stratified into high- (red) and low-score (blue) groups based on subtype-specific CCJ signatures across five subtypes (LumA, LumB, Her2, Basal, and Claudin-low). **(B)** Forest plot summarizing multivariate Cox regression results for the METABRIC dataset, adjusted for patient age. **(C)** Kaplan-Meier curves for the TCGA dataset across four available subtypes (LumA, LumB, Her2, and Basal). **(D)** Forest plot summarizing multivariate Cox regression results for the TCGA dataset, adjusted for patient age. Hazard ratios (HRs) and 95% confidence intervals (CIs) indicate the risk associated with high CCJ signature scores relative to low scores. *P*-values were calculated using the log-rank test (Kaplan-Meier) and the Wald test (Cox regression). In the Kaplan-Meier plots, red text indicates statistical significance (*p* < 0.05).

Breast cancer is a highly heterogeneous disease in which diverse molecular pathways contribute to tumor development, progression, and treatment response. Reliance on individual gene expression is often insufficient to capture the complexity of tumor biology. Gene expression signatures that reflect coordinated activity of multiple genes provide a more robust framework for accurate prognostic and predictive assessment. Building on our previous findings [38], demonstrating the utility of information-theoretic gene signature analysis in breast cancer, we integrated subtype-specific prognostic CCJ genes to construct CCJ signatures for each intrinsic subtype by calculating CCJ signature scores, with the exception of the Normal subtype, which lacked common prognostic genes. The prognostic performance of these CCJ signatures was evaluated, using Kaplan-Meier survival analysis and multivariate Cox proportional hazards models for DSS, adjusting for age. In the METABRIC cohort, Kaplan-Meier analysis demonstrated that patients with high CCJ signature scores consistently exhibited significantly poorer DSS compared with those with low scores across all subtypes (LumA, *p* < 0.001; LumB, *p* < 0.001; Her2, *p* < 0.001; Basal, *p* = 0.013; Claudin-low, *p* < 0.001) (Fig 4A). Multivariate Cox analysis confirmed these findings, revealing that high CCJ signature scores were independently associated with worse DSS (LumA: hazard ratio [HR] = 2.29, 95% confidence interval [CI] = 1.63–3.22, *p* < 0.001; LumB: HR = 2.15, 95% CI = 1.57–2.96, *p* < 0.001; Her2: HR = 2.17, 95% CI = 1.41–3.35, *p* < 0.001; Basal: HR = 1.75, 95% CI = 1.12–2.74, *p* = 0.013; Claudin-low: HR = 2.69, 95% CI = 1.50–4.82, *p* < 0.001) (Fig 4B). Consistent results were observed in the TCGA cohort. Kaplan-Meier analysis showed significantly poorer DSS among patients with high CCJ signature scores across all subtypes (LumA, *p* = 0.002; LumB, *p* = 0.005; Her2, *p* < 0.001; Basal, *p* < 0.001) (Fig 4C). Multivariate Cox proportional hazards analysis also demonstrated that high CCJ signature scores independently predicted worse DSS in each subtype (LumA: HR = 3.86, 95% CI = 1.55–9.58, *p* = 0.004; LumB: HR = 5.19, 95% CI = 1.47–18.26, *p* = 0.010; Her2: HR = 8.10, 95% CI = 2.18–30.07, *p* = 0.002; Basal: HR = 8.58, 95% CI = 3.01–24.49, *p* < 0.001) (Fig 4D). The predictive accuracy of these signatures was further supported by ROC analysis (S2 Table). Collectively, these results indicate that high CCJ signature expression is associated with worse clinical outcomes across intrinsic subtypes.

### Validation of CCJ signatures in the SCAN-B dataset

**Fig 5:**
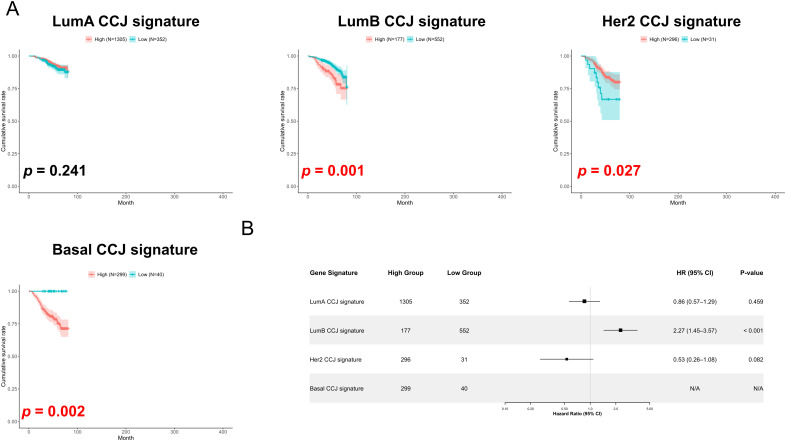
Independent validation of the prognostic value of CCJ signatures in the SCAN-B dataset. Kaplan-Meier survival analysis and multivariate Cox proportional hazards models were performed to validate the prognostic utility of CCJ signatures in the SCAN-B cohort. **(A)** Kaplan-Meier curves for OS across LumA, LumB, Her2, and Basal subtypes. Patients were stratified into high- (red) and low-score (blue) groups based on the subtype-specific CCJ signatures. **(B)** Forest plot summarizing the multivariate Cox regression results adjusted for patient age. Hazard ratios (HRs) and 95% confidence intervals (CIs) are shown. Multivariate analysis for the Basal subtype could not be performed (indicated as N/A) due to the absence of events in the low-score group. *P*-values were calculated using the log-rank test (Kaplan-Meier) and the Wald test (Cox regression). In the Kaplan-Meier plots, red text indicates statistical significance (*p* < 0.05).

To independently validate the prognostic value of the CCJ signatures, we performed identical analyses using the SCAN-B dataset, which was not used during the initial gene extraction. Distinct subtype-specific patterns were observed. In the LumA subtype, no significant prognostic difference was detected between patients with high and low CCJ signature scores, as assessed by Kaplan-Meier analysis (*p* = 0.241) and multivariate Cox proportional hazards modeling (HR = 0.86, 95% CI = 0.57–1.29, *p* = 0.459) (Fig 5A and 5B). In contrast, the Her2 subtype showed an inverse pattern: patients with low CCJ signature scores exhibited significantly poorer survival in Kaplan-Meier analysis (*p* = 0.027). However, this association did not remain statistically significant after adjustment in the multivariate Cox model (HR = 0.53, 95% CI = 0.26–1.08, *p* = 0.082) (Fig 5A and 5B). Importantly, in the LumB subtype, patients with high CCJ signature scores were strongly associated with poor prognosis in the Kaplan-Meier analysis (*p* = 0.001) and multivariate Cox model (HR = 2.27, 95% CI = 1.45–3.57, *p* < 0.001) compared with those with low scores, which is consistent with the results from the discovery cohorts (Fig 5A and 5B). Similarly, in the Basal subtype, high CCJ signature scores were strongly associated with poor prognosis in the Kaplan-Meier analysis (*p* = 0.002) (Fig 5A). Because no disease-specific deaths occurred in the low-score group, multivariate Cox analysis could not be performed for this subtype. The predictive accuracy of these signatures was further supported by receiver operating characteristic (ROC) analysis (S2 Table). Collectively, these results indicate that high CCJ signature expression is associated with worse clinical outcomes in LumB and Basal tumors. The lack of concordant associations in LumA and Her2 subtypes likely reflects inter-cohort heterogeneity in clinical characteristics or transcriptomic profiling. Overall, these findings provide independent validation of the CCJ signature as a prognostic biomarker, particularly in the LumB and Basal subtypes of breast cancer.

### Histological subtype composition and histology-adjusted prognostic analysis of CCJ signatures

**Fig 6:**
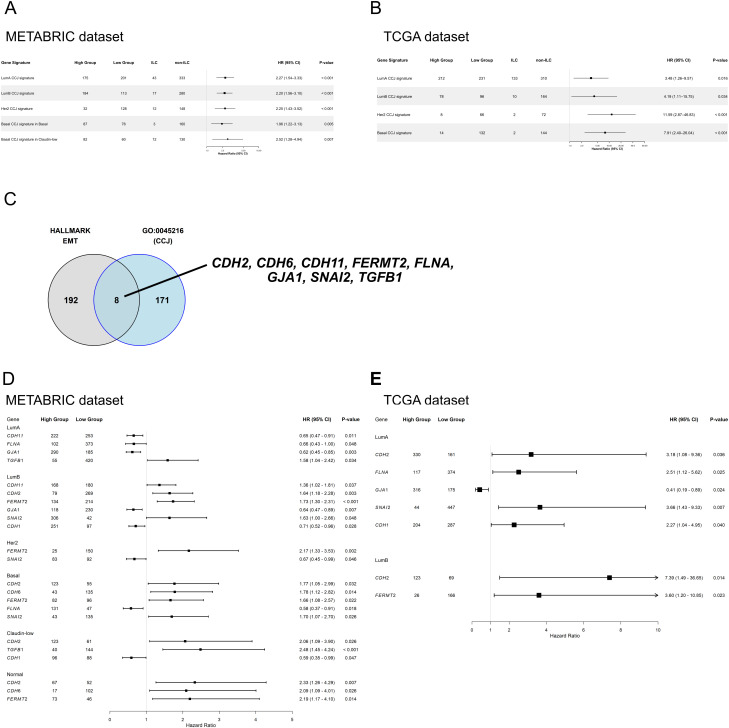
Histology-adjusted prognostic evaluation of CCJ signatures and EMT-related CCJ genes in the METABRIC and TCGA datasets. **(A, B)** Forest plots summarizing multivariable Cox proportional hazards models for subtype-matched CCJ signatures adjusted for age and histological subtype in the METABRIC and TCGA datasets, respectively. **(C)** Venn diagram illustrating the overlap between genes annotated under the Gene Ontology term “cell–cell junction organization” (GO:0045216) and the HALLMARK_EPITHELIAL_MESENCHYMAL_TRANSITION gene set. **(D, E)** Forest plots summarizing multivariable Cox proportional hazards analyses of the overlapping genes and *CDH1* across breast cancer subtypes in the METABRIC and TCGA datasets, respectively. Hazard ratios (HRs) and 95% confidence intervals (CIs) are shown. Only genes significantly associated with poor or favorable prognosis are displayed.

Because histological subtypes may influence the interpretation of the subtype-specific prognostic associations of the CCJ signatures, we performed additional histology-focused analyses in the METABRIC and TCGA cohorts. In particular, invasive lobular carcinoma (ILC), which is pathognomonically associated with loss of E-cadherin [43], was considered a potential confounding factor. First, we summarized the distribution of ILC and non-ILC cases across PAM50 intrinsic subtypes in both cohorts (S1A and S1B Fig). In both datasets, ILC cases were present across subtypes but consistently represented a minority compared with non-ILC cases. Next, we compared CCJ signature scores between ILC and non-ILC tumors within each intrinsic subtype (S1C and S1D Fig). In the METABRIC cohort, a significant difference was observed only in the LumA subtype, in which non-ILC tumors exhibited lower CCJ signature scores than ILC tumors. However, this finding was not reproduced in the TCGA cohort, and no consistent differences in CCJ signature scores between ILC and non-ILC cases were observed across subtypes. Finally, we performed multivariable Cox proportional hazards analyses including ILC status as a covariate (Fig 6A and 6B). Importantly, the prognostic associations of the subtype-matched CCJ signatures remained significant after adjustment for histological subtype, indicating that these associations were not explained solely by histology.

### EMT-related prognostic analysis of CCJ-associated genes

To further examine whether the prognostic relevance of the CCJ signatures could be explained by canonical EMT-related genes, we assessed the overlap between the GO:0045216 “cell–cell junction organization” gene set and the HALLMARK_EPITHELIAL_MESENCHYMAL_TRANSITION gene set from MSigDB [40], and additionally included *CDH1* because of its central biological relevance to EMT (Fig 6C). We then performed subtype-specific multivariable Cox proportional hazards analyses for the overlapping genes and *CDH1* in the METABRIC and TCGA cohorts (Fig 6D and 6E and S3 Table). Although several genes showed significant prognostic associations in individual subtypes, only a limited number were reproducibly associated with prognosis across both discovery cohorts. Specifically, *GJA1* in LumA and *CDH2* and *FERMT2* in LumB showed concordant prognostic associations in both datasets. In contrast, *CDH1* showed prognostic significance only in a subset of analyses and was not reproducibly associated across the two cohorts. These findings indicate that the prognostic relevance captured by the CCJ signatures is not recapitulated by E-cadherin alone and is not fully explained by canonical EMT-related genes.

### Leave-one-gene-out sensitivity analysis of the CCJ signature

To evaluate whether the prognostic performance of each CCJ signature was driven by any single constituent gene, we performed a leave-one-gene-out sensitivity analysis in the METABRIC, TCGA, and SCAN-B cohorts (S2 Fig). In the discovery cohorts, omission of individual genes produced only modest changes in hazard ratios, and the prognostic associations of the subtype-matched CCJ signatures were largely preserved across subtypes (S2A and S2B Fig). These findings indicate that the prognostic relevance of the CCJ signatures in the discovery cohorts was not driven by any single gene, but rather reflected a collective signal. In the SCAN-B cohort, LumB showed the most stable pattern, whereas LumA and Her2 showed more limited or inconsistent effects. For the Basal subtype in the SCAN-B cohort, the original model is not shown because it was identical to the primary validation model and was not estimable owing to the absence of death events in the low-score group. In contrast, in leave-one-gene-out models that became estimable, hazard ratios varied substantially, suggesting that this subtype was strongly influenced by the limited number of events in the validation cohort rather than by the contribution of any specific gene (S2C Fig). Overall, these findings support that the principal prognostic associations of the CCJ signatures, particularly in the discovery cohorts and in the LumB validation analysis, were not driven by any single gene but instead reflected a robust collective signal.

### Gene-number downsampling analysis of the CCJ signature

To determine whether a minimum number of genes was required for each CCJ signature to remain informative, we performed a gene-number downsampling analysis in the METABRIC, TCGA, and SCAN-B cohorts (S3 Fig). Reduced signatures were assembled according to the subtype-specific integrated rank table (S4 Table), such that genes with more consistently higher ranks across the discovery cohorts were preferentially retained in the top 7-, top 5-, and top 3-gene sets. In the discovery cohorts, the prognostic associations of most CCJ signatures were largely preserved as gene number was reduced. In METABRIC, the LumA, LumB, Her2, and Claudin-low signatures remained significantly associated with prognosis even in smaller gene sets. For the Basal signature, reduced gene sets yielded similar hazard ratios, although statistical significance became borderline in some versions. In TCGA, all evaluated signatures retained significant prognostic associations across the downsampled gene sets. In the SCAN-B cohort, the LumB signature remained significantly associated with prognosis across all evaluated gene sets, with hazard ratios of 2.27 (95% CI = 1.45–3.57, *p* < 0.001) for the original signature, 2.27 (95% CI = 1.40–3.69, *p* < 0.001) for the top 7-gene set, 1.67 (95% CI = 1.08–2.58, *p* = 0.021) for the top 5-gene set, and 1.85 (95% CI = 1.19–2.86, *p* = 0.006) for the top 3-gene set (S3C Fig). Although prognostic information was retained in the reduced gene sets, the original and top 7-gene versions showed the most stable performance, suggesting that retaining a larger number of genes may be advantageous for more robust prognostic stratification. For the Basal subtype, the original model could not be estimated because no death events occurred in the low-score group, whereas the downsampled gene sets showed significant associations with prognosis. These findings indicate that no single universal minimum gene number could be defined across all subtype-cohort combinations. Nevertheless, the overall preservation of prognostic associations across multiple reduced gene sets suggests that the CCJ signatures retain prognostic relevance, while the more stable performance of the original or moderately reduced signatures indicates that larger gene sets may be advantageous for more robust stratification.

### Recurrence risk stratification using CCJ signatures

**Fig 7:**
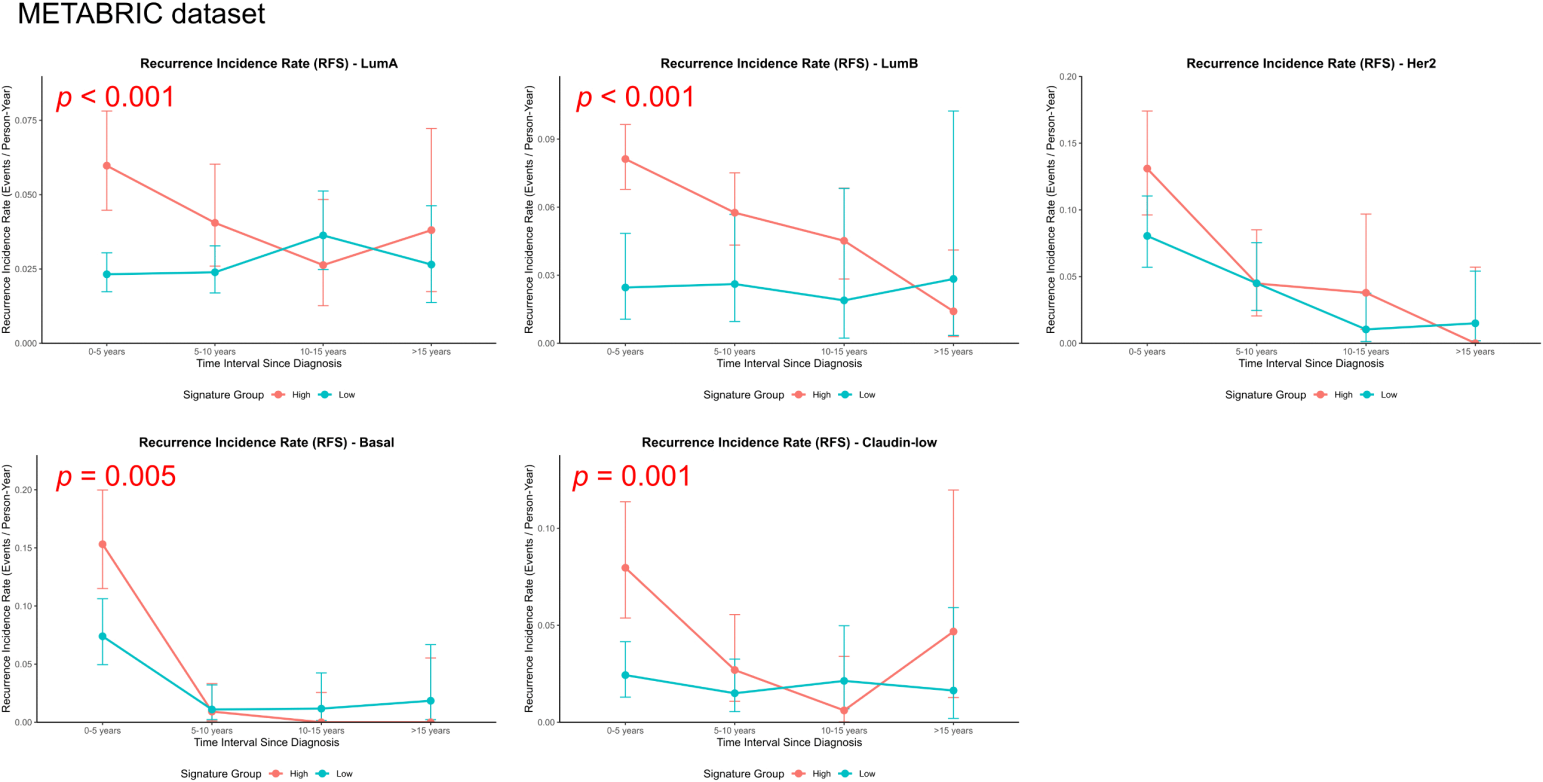
Recurrence risk associated with the CCJ signature assessed by person-year analysis in METABRIC dataset. Recurrence incidence rates (events per person-year) were calculated for patients stratified into high- (red) and low-score (blue) CCJ signature groups. **(A)** METABRIC dataset assessed over 0–5, 5–10, 10–15, and >15 year intervals. *P*-values derived from Poisson tests with Holm correction indicate statistical significance (*p* < 0.05) or trends. Error bars indicate 95% confidence intervals (CIs).

To evaluate recurrence risk with CCJ signature expression, recurrence incidence rates per person-year were calculated across defined time intervals (0–5, 5–10, 10–15, and >15 years for METABRIC; 0–5, 5–10, and >10 years for TCGA) (Figs 7 and S4 Fig and S5 Table). In the METABRIC dataset, patients with high CCJ signature scores consistently exhibited significantly higher recurrence rates during the first five years across LumA (*p* < 0.001), LumB (*p* < 0.001), Basal (*p* = 0.005), and Claudin-low (*p* = 0.001) subtypes. In the LumB subtype, recurrence rates in the high-score group remained elevated during the 5–10 and 10–15 year intervals, although these differences did not reach statistical significance (Fig 7). Consistent trends were observed in the TCGA cohort using disease-free survival (DFS) as the endpoint. In this dataset, the LumB subtype showed a trend towards higher event rates in the high CCJ signature group during the first 0–5 years (*p* = 0.051), and a similar pattern was observed in the Basal subtype (*p* = 0.091) (S4 Fig). Together, these findings highlight the ability of the CCJ signature to identify patients at increased risk of early recurrence (0–5 years), particularly within aggressive and luminal subtypes, suggesting its potential use in identifying patients vulnerable to early treatment failure.

### Differential gene expression associated with CCJ signature scores

**Fig 8:**
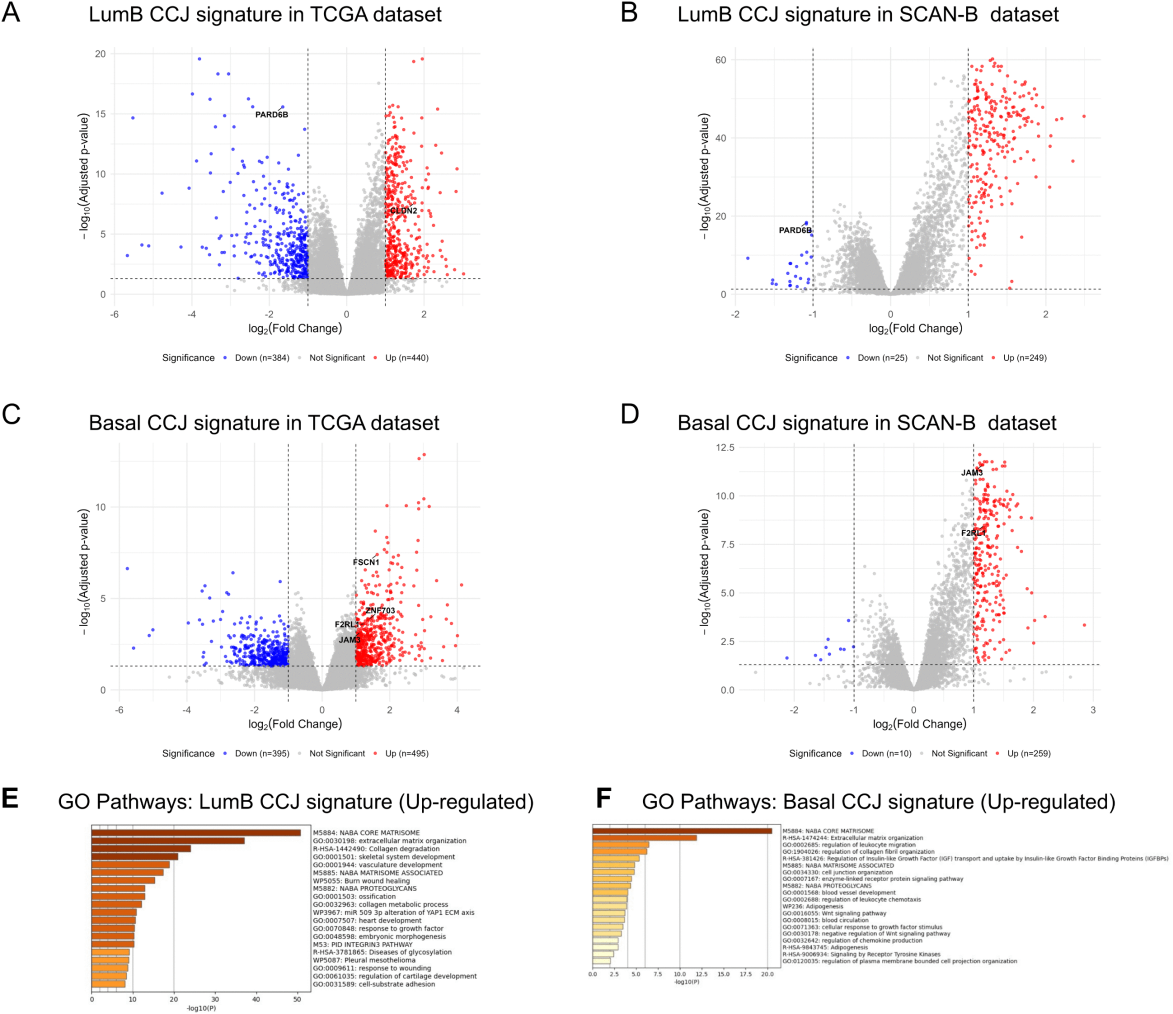
Differential gene expression and functional enrichment analysis associated with high CCJ signature scores. Volcano plots illustrate DEG comparing high- and low-CCJ signature score groups in the TCGA and SCAN-B datasets. Red dots represent significantly upregulated genes, blue dots represent significantly downregulated genes, and grey dots represent non-significant genes. Genes included in the CCJ signatures are annotated on the volcano plots with labels connected to the corresponding dots by leader lines. **(A)** LumB subtype in the TCGA dataset. **(B)** LumB subtype in the SCAN-B dataset. **(C)** Basal subtype in the TCGA dataset. **(D)** Basal subtype in the SCAN-B dataset. **(E, F)** Bar plots displaying the top enriched Gene Ontology (GO) terms and pathways for the genes that were commonly upregulated in both datasets for **(E)** the LumB CCJ signature and **(F)** the Basal CCJ signature.

To further characterize the molecular properties associated with high CCJ signature expression, patients were stratified into high- and low-score groups, and differentially expressed gene (DEG) analyses were performed, using RNA-seq data from the TCGA and SCAN-B cohorts (Figs 8A–8D and S5). These cohorts were selected because both employed RNA-seq-based profiling and shared PAM50 subtype annotation, with exclusion of the Claudin-low subtype. DEG analysis revealed distinct subtype-specific transcriptional differences between high- and low-score tumors. For the LumB CCJ signature, 440 genes were upregulated and 384 were downregulated in the TCGA cohort (Fig 8A), whereas 249 genes were upregulated and 25 were downregulated in the SCAN-B cohort (Fig 8B). Among these, 93 genes were consistently upregulated and four genes (*COX6C*, *DIO1*, *PHGR1*, *PARD6B*) were consistently downregulated across both TCGA and SCAN-B datasets (S6 Table). For the Basal CCJ signature, the TCGA cohort exhibited 495 upregulated and 395 downregulated genes (Fig 8C), while the SCAN-B cohort exhibited 259 upregulated and 10 downregulated genes (Fig 8D). Of these, 60 genes were consistently upregulated and four genes (*FABP7*, *KLHDC7B*, *SPIB*, *UBD*) were consistently downregulated across both cohorts (S6 Table). Given the substantial number of commonly upregulated genes in the LumB (n = 93) and Basal (n = 60) signatures, Gene Ontology (GO) enrichment analysis was performed to elucidate the biological processes associated with high CCJ signature expression (Fig 8E and 8F). Both signatures showed strong enrichment for extracellular matrix (ECM)-related pathways. In the LumB CCJ signature, significantly enriched terms included “NABA CORE MATRISOME,” “extracellular matrix organization,” and “collagen degradation.” Similarly, the Basal CCJ signature was enriched for “NABA CORE MATRISOME,” “extracellular matrix organization,” and “regulation of collagen fibril organization.” In addition to ECM-related processes, the Basal signature was enriched for “cellular response to growth factor stimulus”.

To further assess the representation and differential expression of CCJ signature genes, we evaluated whether each gene was (i) present in the expression matrix, (ii) retained after expression filtering, and (iii) identified as a significant DEG under the predefined criteria. In the TCGA cohort, 35 of 39 signature genes were detected in the DESeq2 results, whereas 29 of 39 genes were detected in the SCAN-B limma results (S7 Table). Among these, only a subset met the stringent DEG criteria (|log_2_ fold change| > 1 and *p* < 0.05). Notably, all signature genes identified as significant DEGs exhibited a consistent direction of effect relative to the CCJ signature definition, supporting the biological coherence of the signatures.

### Expression pattern of CCJ signature genes in cells of breast cancer tissues

**Fig 9:**
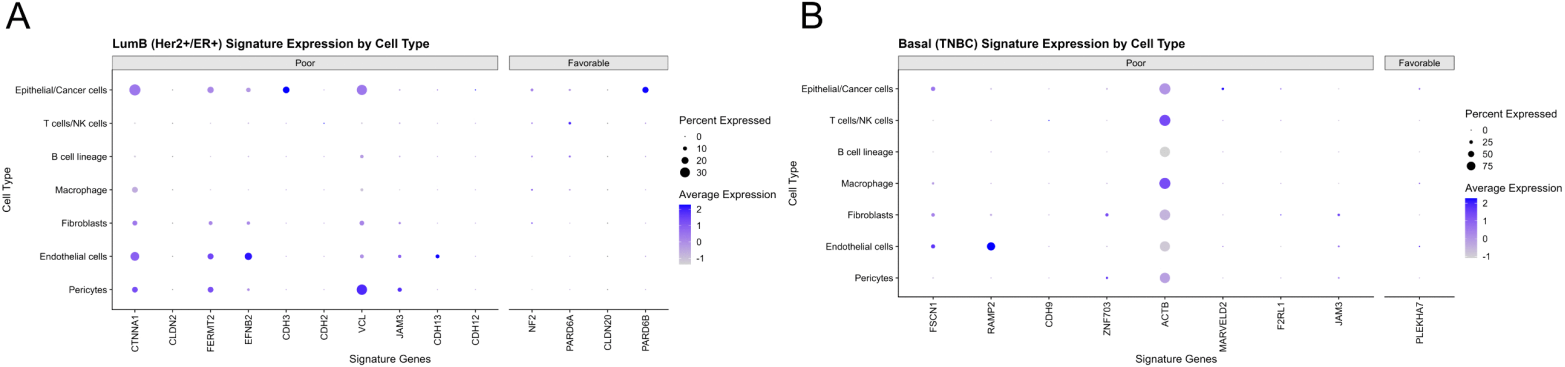
Expression pattern of CCJ signature genes in cells of breast cancer tissues. Dot plots visualize expression patterns of genes constituting the **(A)** LumB CCJ signature in an Her2 + /ER+ patient (n = 1) and **(B)** Basal CCJ signature in Triple-Negative Breast Cancer (TNBC) patients (n = 7), from the GSE176078 dataset. The x-axis lists the signature genes, and the y-axis represents identified cell types. Dot size indicates the percentage of cells expressing the gene (“Percent Expressed”), while color intensity represents the average expression level (“Average Expression”).

Given the pronounced prognostic relevance of CCJ signatures in LumB and Basal subtypes, we subsequently examined the predominant cell populations expressing genes constituting these subtype-specific signatures. Using single-cell RNA-seq data from breast cancer patients (GSE176078), we analyzed expression patterns of genes in the LumB CCJ signature using data from an Her2 + /ER+ tumor (n = 1) as a representative LumB case. Likewise, because Basal tumors largely overlap with TNBC, we analyzed expression of genes in the Basal CCJ signature across TNBC patients (n = 7) (Figs 9 and S6). Within the LumB CCJ signature, *PARD6B* (favorable prognostic gene) and *CDH3* (poor prognostic gene) were predominantly expressed in epithelial/cancer cell populations (Figs 9A and S6). In contrast, *CTNNA1* (poor prognostic gene) displayed broader expression across multiple cell types, including epithelial/cancer cells, macrophages, fibroblasts, endothelial cells, and pericytes (Figs 9A and S6). For the Basal CCJ signature, *MARVELD2* (poor prognostic gene) was mainly expressed in epithelial/cancer cells, whereas *RAMP2* (poor prognostic gene) showed predominant expression in vascular endothelial cells (Figs 9B and S6). Additionally, *ZNF703* (poor prognostic gene) was mainly expressed in stromal components, specifically in fibroblasts and pericytes (Figs 9B and S6). These observations indicate that CCJ signature genes exhibit heterogeneous cell type-specific expression patterns, reflecting contributions from both tumor-intrinsic epithelial cells and compartments of the tumor microenvironment.

## Discussion

In this study, we systematically analyzed genes involved in CCJ organization across intrinsic breast cancer subtypes and identified distinct subtype-specific prognostic signatures. These CCJ signatures demonstrated consistent prognostic value across multiple independent cohorts (METABRIC, TCGA, SCAN-B), with particularly robust associations observed in LumB and Basal tumors. DEG analyses revealed subtype-specific transcriptional programs associated with high-CCJ signature scores while single-cell RNA-seq analyses provided preliminary evidence that selected CCJ genes are expressed in distinct cellular compartments. Together, these findings suggest that both tumor-intrinsic properties and components of tumor microenvironment contribute to the prognostic relevance of CCJ signatures. Collectively, our results provide new insight into how coordinated CCJ-related gene expression captures subtype-specific tumor biology and clinical behavior in breast cancer. The prognostic implications of cell–cell adhesion molecules in cancer have long been paradoxical. Classical junctional proteins, including E-cadherin, claudins, and occludin, have been implicated in both tumor suppression and malignant progression, depending on molecular and cellular context [24,27–29]. This heterogeneity has hindered the development of a unified understanding of how CCJ-related pathways influence cancer progression. By integrating large-scale, multi-cohort transcriptomic datasets and constructing subtype-specific CCJ gene expression signatures, our study offers a systems-level perspective demonstrating that, despite the diverse and sometimes opposing functions of individual junctional components, the coordinated expression of CCJ-related genes follows biologically meaningful, subtype-dependent prognostic patterns. This framework provides conceptual reconciliation for previously inconsistent findings in CCJ biology.

Histological subtype, particularly invasive lobular carcinoma (ILC), is an important consideration when interpreting CCJ-related prognostic signals, as loss of E-cadherin is pathognomonic of ILC and directly linked to disruption of cell–cell adhesion. In the present study, although a significant difference in CCJ signature scores between ILC and non-ILC tumors was observed in the LumA subtype of the METABRIC cohort, this finding was not reproduced in the corresponding TCGA subgroup. Moreover, the prognostic associations of the subtype-matched CCJ signatures remained significant after adjustment for histological subtype in both cohorts. These findings suggest that, although lobular histology may contribute to tumor heterogeneity in specific contexts, it does not fully account for the subtype-specific prognostic associations of the CCJ signatures. Rather, these associations appear to reflect broader subtype-dependent junctional programs that are not reducible to histological subtype alone. Consistent with this observation, our additional analyses further indicated that the prognostic associations of the CCJ signatures are not explained solely by canonical epithelial–mesenchymal transition (EMT)-related genes. Although EMT and E-cadherin dysregulation are central to breast cancer progression, only a limited number of EMT-related CCJ-associated genes were reproducibly associated with prognosis across both cohorts. Specifically, *GJA1* in LumA and *CDH2* and *FERMT2* in LumB showed concordant associations in both datasets, whereas *CDH1* was significant only in a subset of analyses and did not show reproducible prognostic associations. These findings suggest that the CCJ signatures are not recapitulated by E-cadherin alone and are not fully accounted for by canonical EMT-related programs. Instead, they appear to capture a broader biological framework encompassing junctional organization and microenvironmental remodeling, consistent with the GO analysis of subtype-specific DEGs and the observed directional concordance of signature genes. Because the CCJ signature assigns equal weight to each constituent gene, we further examined whether the observed prognostic associations were disproportionately influenced by any single gene. Leave-one-gene-out analyses demonstrated that omission of individual genes did not materially alter the overall direction of the prognostic effect in the discovery cohorts, indicating that the CCJ signatures reflect a collective signal rather than dependence on any single gene. In the SCAN-B cohort, the LumB subtype showed the most stable pattern, whereas LumA and Her2 exhibited more limited or inconsistent effects, likely reflecting weaker baseline associations in these subtypes. For the Basal subtype, interpretation was limited because the original model was not estimable owing to the absence of events in the low-score group, and variability observed in estimable models was likely driven by limited event numbers rather than gene-specific effects.

The gene-number downsampling analysis further supported the robustness of the CCJ signatures. In the discovery cohorts, prognostic associations were largely preserved as gene number decreased. In the SCAN-B cohort, the LumB signature remained significantly associated with prognosis across all reduced gene sets, although the original and top 7-gene versions showed the most stable performance. For the Basal subtype, the original model was not estimable, whereas reduced gene sets yielded significant associations, likely reflecting improved model estimability after gene set reduction rather than increased robustness. Overall, these findings indicate that no single universal minimum gene number can be defined across all subtype–cohort combinations. Nevertheless, the preservation of prognostic associations across reduced gene sets suggests that the CCJ signatures retain overall prognostic associations, while the more stable performance of larger gene sets indicates that retaining a moderate number of genes may be advantageous for robust stratification. Our subtype-stratified analysis offers mechanistic insights into how adhesion-related molecules influence tumor plasticity and progression. We did not identify any CCJ-related genes that were consistently associated with prognosis in the Normal subtype across both the METABRIC and TCGA cohorts. This lack of reproducible associations suggests that CCJ-related pathways may play a limited or context-dependent role in tumor progression within this subtype, potentially reflecting distinct biological characteristics of this subtype. Collectively, these findings indicate that the biological and clinical relevance of CCJ-related genes in the Normal subtype remains unclear and warrants further investigation.

In the LumA CCJ signature, high expression of *INAVA*, a SMAD3-dependent transforming growth factor-β (TGF-β) target gene, was linked to poor prognosis. INAVA has been implicated in breast cancer stem-like properties and metastatic potential through upregulation of ELF5 and downregulation of GATA3, suggesting a possible role of TGF-β–SMAD3–ELF5 circuitry in LumA tumor plasticity [44,45]. In the LumB CCJ signature, *CLDN2* has been associated with liver metastasis, while *FERMT2* (*Kindlin-2*) has been implicated in tumor progression through macrophage-dependent signaling pathways involving TGF-β and colony-stimulating factor 1 (CSF-1) [46–48]. These observations align with our pathway enrichment analyses, which identified ECM remodeling and tumor–stroma communication as prominent features of LumB tumors with high CCJ signature expression. For the Her2 CCJ signature, high *CTNNB1* expression was associated with poor prognosis. However, the clinical impact of β-catenin is known to be strongly influenced by its subcellular localization [49], indicating that transcript abundance alone may be insufficient to capture the functional consequences of CCJ-related signaling in Her2-positive disease. Notably, therapeutic Her2 inhibition has been reported to induce compensatory activation of Notch-1 signaling, contributing to treatment resistance [50,51]. Consequently, low expression of *NUMB*, a Notch suppressor, may compromise treatment efficacy. This mechanism is consistent with our finding that higher *NUMB* expression correlates with favorable prognosis and warrants further investigation in the context of resistance pathways. In the Basal CCJ signature, *RAMP2*, which was predominantly expressed in vascular endothelial cells, was associated with poor prognosis. The adrenomedullin–RAMP2 signaling axis is known to stimulate angiogenesis, particularly under macrophage-mediated regulation [52,53]. In contrast, *MARVELD2* (*tricellulin*), expressed primarily in epithelial/cancer cells, has been linked to aggressive phenotypes across multiple tumor types, and its association with protease-activated receptor 2 (PAR2)-related signaling suggests convergence on pathways that promote proliferation [54–56]. Importantly, our single-cell analyses revealed that CCJ genes contributing to prognostic signals originate from tumor epithelial compartments (e.g., *PARD6B*, *CDH3*, *MARVELD2*), while others exhibited enrichment in endothelial or stromal populations (e.g., *RAMP2*, *ZNF703*), or displayed broad expression patterns across the tumor microenvironment (e.g., *CTNNA1*). Although these observations are based on a limited number of single-cell datasets and require further validation, they suggest that subtype-specific CCJ signatures may integrate signals derived from distinct cellular sources. Consistent with this interpretation, CCJ signatures not only stratified patient prognosis but also reflected differences in cellular composition, offering mechanistic insight into subtype-specific tumor biology. Functional enrichment analyses further supported shared biological programs, with both LumB and Basal signatures showing consistent enrichment for ECM remodeling pathways, including matrisome activation and collagen turnover. These findings suggest that high CCJ signature expression reflects microenvironmental restructuring that may facilitate tumor invasion. In Basal tumors, additional enrichment of growth factor response frameworks aligned with aggressive phenotypes and resistance biology.

Recent work by Esquivel et al. [57] introduced the AdhesionScore, a prognostic framework based on a broad definition of cell adhesion encompassing both cell–cell and cell–extracellular matrix interactions. In contrast, the present study focuses specifically on genes associated with cell–cell junction organization (GO:0045216), thereby targeting processes related to junctional structure and remodeling rather than the broader adhesion landscape. Furthermore, whereas AdhesionScore was developed as a general prognostic model across breast cancer, our CCJ signatures were constructed in a subtype-specific manner using genes with reproducible prognostic associations within individual intrinsic subtypes. Notably, there was no direct overlap between the genes included in AdhesionScore and those in our CCJ signatures, suggesting that these approaches capture related but conceptually and analytically distinct biological signals. Taken together, these findings suggest that CCJ-based signatures may complement existing adhesion-based frameworks by providing additional subtype-specific insights into breast cancer prognosis.

Together, the functional enrichment and single-cell analyses support a model in which CCJ-associated pathways link epithelial junctional signaling with stromal interactions, thereby contributing to subtype-specific tumor progression and potentially affecting therapeutic response [58,59]. These biological characteristics have direct translational implications. From a clinical perspective, CCJ signatures represent a biologically distinct axis for patient stratification compared with currently implemented multigene assays. Whereas assays such as Oncotype DX, MammaPrint, and EndoPredict refine recurrence risk prediction in hormone receptor–positive breast cancer using predefined gene modules, the CCJ signatures described here capture processes related to intercellular adhesion, ECM remodeling, and tumor–microenvironment interactions. Our recurrence risk analyses demonstrated that high CCJ signature scores are also associated with an increased incidence of early recurrence (0–5 years), particularly in aggressive subtypes. Rather than replacing existing assays, CCJ signatures may therefore complement current risk stratification frameworks by highlighting microenvironment and adhesion-driven vulnerabilities, especially in LumB and Basal tumors, where existing tools have recognized limitations.

Several limitations of this study should be acknowledged. First, the use of retrospective datasets introduces heterogeneity in follow-up duration, sequencing platforms, and clinical management. Second, the CCJ signature score assigns equal weight to each constituent gene and therefore may not fully account for differences in biological importance or potential redundancy among CCJ-related genes. Third, spatial resolution was limited. Although our single-cell analyses provided initial mechanistic insight into the cellular origins of CCJ-related prognostic signals, broader single-cell profiling, spatial transcriptomics, and proteomic imaging approaches will be required to validate these observations and more precisely define the spatial and cellular context of CCJ gene expression. Additionally, functional validation of key signaling axes identified in this study, such as adrenomedullin–RAMP2 pathway or Kindlin-2–TGF-β-mediated interactions, will be necessary to establish causal relationships. Despite these limitations, our integrative analyses demonstrate that CCJ-related gene signatures capture meaningful aspects of tumor heterogeneity and prognosis, providing a foundation for future studies aimed at identifying therapeutically actionable pathways linked to ECM remodeling and vascular or endothelial involvement.

In summary, our findings highlight CCJ signatures as biologically meaningful determinants of breast cancer heterogeneity and clinical outcome. By linking CCJ-associated transcriptional programs to microenvironmental remodeling and cellular source diversity, this study provides a framework for refining subtype-specific risk stratification and for developing therapeutic strategies targeting adhesion-associated pathways.

## Supporting information

S1 Fig Histological subtype composition and comparison of CCJ signature scores by histology in the METABRIC and TCGA datasets. (A, B) Histological subtype composition within each intrinsic subtype in the METABRIC and TCGA datasets, respectively. (C, D) Comparison of CCJ signature scores between invasive lobular carcinoma (ILC) and non-ILC cases within each subtype in the METABRIC and TCGA datasets, respectively. In panel (C), * indicates *p* < 0.05.
(TIFF)

S2 Fig Leave-one-gene-out sensitivity analysis of subtype-specific CCJ signatures. Leave-one-gene-out sensitivity analyses were performed to evaluate the robustness of the subtype-specific CCJ signatures. Hazard ratios (HRs) and 95% confidence intervals (CIs) from age-adjusted multivariable Cox proportional hazards models are shown for the original signature (red) and for signatures recalculated after omission of one gene at a time (black). (A) METABRIC dataset. (B) TCGA dataset. (C) SCAN-B dataset. The vertical dashed line indicates HR = 1, and the red dotted line indicates the HR of the original signature. In the METABRIC dataset, the Basal CCJ signature was also evaluated in the Claudin-low subtype.
(TIFF)

S3 Fig Gene-number downsampling analysis of CCJ signatures in METABRIC, TCGA, and SCAN-B datasets. (A, B, C) Forest plots summarizing age-adjusted multivariable Cox proportional hazards models for the original and downsampled CCJ signatures in the METABRIC, TCGA, and SCAN-B datasets, respectively. Downsampled signatures were generated by reducing the number of genes in each subtype-specific CCJ signature. Hazard ratios (HRs) and 95% confidence intervals (CIs) are shown for each signature version. In METABRIC, the Basal CCJ signature was additionally evaluated in the Claudin-low subtype. In SCAN-B, only genes with observed expression values were retained.
(TIFF)

S4 Fig Recurrence risk associated with the CCJ signature assessed by person-year analysis in TCGA dataset. Recurrence incidence rates (events per person-year) were calculated for patients stratified into high- (red) and low-score (blue) CCJ signature groups. (A) TCGA dataset (DFS events) assessed over 0–5, 5–10, and >10 year intervals. Error bars indicate 95% confidence intervals (CIs). *P*-values (Poisson test with Holm adjustment) indicating significance (*p* < 0.05) or trends are displayed.
(TIFF)

S5 Fig Differential gene expression (DEG) analysis associated with high CCJ signature scores in LumA and Her2 subtypes. Volcano plots illustrate DEG comparing high- and low-CCJ signature score groups in the TCGA and SCAN-B datasets. Red dots represent significantly upregulated genes, blue dots represent significantly downregulated genes, and grey dots represent non-significant genes. Genes included in the CCJ signatures are annotated on the volcano plots with labels connected to the corresponding dots by leader lines. (A) LumA subtype in the TCGA dataset. (B) LumA subtype in the SCAN-B dataset. (C) Her2 subtype in the TCGA dataset. (D) Her2 subtype in the SCAN-B dataset.
(TIFF)

S6 Fig UMAP visualization of cell type clusters and CCJ signature gene expression in single-cell RNA-seq data. (A, B) Uniform Manifold Approximation and Projection (UMAP) plots showing annotated cell-type clusters for (A) an Her2 + /ER+ patient (representing the LumB context) and (B) TNBC patients (representing the Basal context) from the GSE176078 dataset. Colors correspond to the identified cell types. (C–F) Feature plots displaying the expression distribution of CCJ signature genes projected onto the UMAP coordinates. Color intensity (purple) represents gene expression levels. (C) Poor prognosis genes within the LumB CCJ signature. (D) Favorable prognosis genes within the LumB CCJ signature. (E) Poor prognosis genes within the Basal CCJ signature. (F) Favorable prognosis genes within the Basal CCJ signature.
(TIFF)

S1 Table List of genes annotated to the Gene Ontology term “cell–cell junction organization” (GO:0045216) and EMT-related genes included in the overlap analysis. This table lists the 179 genes included in the initial screening based on GO:0045216, as well as the genes overlapping with the HALLMARK_EPITHELIAL_MESENCHYMAL_TRANSITION gene set used for the EMT-related analysis.
(XLSX)

S2 Table Receiver operating characteristic (ROC) analysis of CCJ signatures. The table presents the Area Under the Curve (AUC), sensitivity, and specificity values for CCJ signatures across different subtypes in the METABRIC, TCGA, and SCAN-B cohorts.
(XLSX)

S3 Table Complete results of multivariable Cox proportional hazards analysis for EMT-related CCJ genes in the METABRIC and TCGA datasets. The table presents hazard ratios (HRs), 95% confidence intervals (CIs), and *p* values for all genes included in the EMT-related prognostic analysis, namely the overlapping genes between the Gene Ontology term “cell–cell junction organization” (GO:0045216) and the HALLMARK_EPITHELIAL_MESENCHYMAL_TRANSITION gene set, together with *CDH1*, across breast cancer subtypes in the METABRIC and TCGA datasets. This table includes all analyzed genes, regardless of statistical significance.
(XLSX)

S4 Table Subtype-specific integrated rank table of CCJ signature genes used for gene-number downsampling analysis. Genes were prioritized within each subtype using prognostic ranking scores derived from the METABRIC and TCGA discovery cohorts. Cohort-specific ranks were integrated to generate a mean rank and final order within each subtype. For the Basal context, METABRIC Basal and Claudin-low statistics are shown separately. Ranking scores for each cohort are provided. The columns Selected_top7, Selected_top5, and Selected_top3 indicate whether each gene was retained in the corresponding reduced signature for the downsampling analyses.
(XLSX)

S5 Table Detailed results of person-year recurrence risk analysis. The table summarizes the number of events, total person-years, incidence rates, and incidence rate ratios (IRR) with 95% confidence intervals for each time interval in the METABRIC and TCGA datasets.
(XLSX)

S6 Table List of consistently differentially expressed genes (DEGs) identified in both TCGA and SCAN-B datasets. This table lists the genes that were significantly and consistently upregulated or downregulated in high-CCJ signature tumors across both TCGA and SCAN-B datasets.
(XLSX)

S7 Table Representation and differential expression status of CCJ signature genes in the TCGA and SCAN-B cohorts. This table summarizes whether each subtype-specific CCJ signature gene was present in the expression matrix, retained for DEG analysis, and differentially expressed between the CCJ signature high- and low-score groups in the TCGA and SCAN-B cohorts. Signature_class indicates whether the gene was classified as poor or favorable in the subtype-specific CCJ signature. Expected_direction indicates the expected direction of differential expression based on the signature class. Direction_in_high_vs_low indicates the observed direction of differential expression in the high-score group relative to the low-score group. direction_match indicates whether the observed direction was consistent with the expected direction.
(XLSX)

S1 File R scripts and input files for the main analyses. This ZIP archive contains R scripts and lightweight input files used for the main analyses, including processed input tables, annotation files, and gene lists.
(ZIP)
